# Short-Term Effects of Fruit Juice Enriched with Vitamin D3, n-3 PUFA, and Probiotics on Glycemic Responses: A Randomized Controlled Clinical Trial in Healthy Adults

**DOI:** 10.3390/metabo13070791

**Published:** 2023-06-25

**Authors:** Nikolaos Zacharodimos, Christina Athanasaki, Stamatia Vitsou-Anastasiou, Olga S. Papadopoulou, Natalia Moniaki, Agapi I. Doulgeraki, George-John E. Nychas, Chrysoula C. Tassou, Emilia Papakonstantinou

**Affiliations:** 1 Laboratory of Dietetics and Quality of Life, Department of Food Science and Human Nutrition, School of Food and Nutritional Sciences, Agricultural University of Athens, 75 Iera Odos, 11855 Athens, Greece; 2 Institute of Technology of Agricultural Products, Hellenic Agricultural Organization “DIMITRA”, Attiki, 14123 Lykovrisi, Greece; 3 Laboratory of Microbiology and Biotechnology of Foods, Department of Food Science and Human Nutrition, School of Food and Nutritional Sciences, Agricultural University of Athens, 75 Iera Odos, 11855 Athens, Greece

**Keywords:** fruit juice, glycemic responses, glycemic index, vitamin D, n-3 fatty acids, probiotics, blood pressure, appetite, healthy adults

## Abstract

This study aimed to determine the glycemic index (GI) of a commercial mixed fruit juice (apple, orange, grape, and pomegranate; FJ) fortified with vitamin D3 or n-3 polyunsaturated fatty acids (PUFA) or probiotics, and their combination, and their effects on glycemic responses and salivary insulin concentrations. In a randomized controlled, double-blind, crossover study, 11 healthy participants (25 ± 2 years; five women; body mass index = 23 ± 1 kg/m^2^) were randomly assigned to receive five types of FJs [vitD (with vitamin D3); n-3 (with n-3 PUFA); probiotics (with *Lacticaseibacillus casei* Shirota and *Lacticaseibacillus rhamnosus* GG); vitD-n-3-probiotics FJ (combination of vitD3-n-3-probiotics), control (regular FJ)], all containing 50 g available carbohydrate, and glucose as reference drink. All FJs provided low GI values (control: 54; vitD3: 52; n-3: 51; probiotics: 50; and vitD-n-3-probiotics combination: 52, on glucose scale). Compared to the FJ control, the enriched FJs produced different postprandial glycemic and insulinemic responses and affected satiety scores. All FJ types, regardless of the added biofunctional ingredients, attenuated postprandial glycemic responses, which may offer advantages to glycemic control.

## 1. Introduction

Health benefits from fresh fruit consumption and ingestion of biofunctional ingredients, such as vitamin D3, n-3 polyunsaturated fatty acids (PUFA), and probiotic strains are well established. However, data from consuming fruit juice (FJ) on health markers have produced inconclusive results. The World Health Organization (WHO) has raised concerns regarding the FJs’ free sugars’ content [1]. However, it should be noted that FJs contain micronutrients and other plant bioactive compounds, such as polyphenols, in amounts similar to those of fruits [2]. The Dietary Guidelines for Americans (2020–2025) allow the consumption of FJs for half of the recommended daily fruit intake [3]. A recent review suggested that FJ consumption at moderate daily intakes (75–224 mL) or higher (up to 500 mL) does not increase the risk of chronic diseases, such as obesity, type 2 diabetes mellitus (T2DM), and cardiovascular diseases (CVD), but on the contrary, may have beneficial effects on vascular function and blood pressure (BP), may induce slower dietary sugars’ gut absorption, and may show anti-inflammatory and antioxidant effects [2]. Results from two meta-analyses of prospective cohorts and randomized controlled clinical trials (RCT) reported no association between consumption of FJ and type 2 diabetes prevalence and no significant effect on glycemic control and blood insulin concentrations [4,5].

The glycemic index (GI) is a well-characterized tool developed to classify foods containing carbohydrates according to time-integrated effects on postprandial glycemia [6]. The GI makes the characterization of the standardized and relative postprandial glucose responses based on an equal amount of available carbohydrates (50 g) and relative to a reference food, typically D-glucose [7,8]. Carbohydrates in high GI (GI ≥ 70, on glucose scale) foods are rapidly digested, absorbed, and metabolized, unlike those in low GI (GI ≤ 55, on glucose scale) foods [7]. Low GI diets may lead to lower postprandial glucose excursions [9]. Consumption of low GI foods has been shown to produce lower glucose fluctuations and glucose variability (from peaks to nadirs) and protection of beta-cell function by lowering oxidative stress, indicating that it may prevent major metabolic defects leading to CVD [10]. Replacement of high GI for low GI foods may also lead to reduced hemoglobin A1C values from −0.2% up to −0.5% [11,12,13], offering some improvement in glycemic control [13,14]. Juices are considered a medium to high GI food [15]. It has been suggested that the fructose to glucose ratio, but not the fiber content, of FJs is inversely associated with GI, postprandial glucose and insulin responses [16].

Moreover, the effects of biofunctional ingredients, such as n-3 PUFA, vitamin D3, or probiotics on appetite are not well understood and inconclusive. It is well known that the physiological state of hunger is regulated by secretion of satiety hormones. Leptin is one of the hormones known to regulate appetite, reducing food intake and accelerating energy expenditure [17]. It has also been suggested that the beverage calorie content and inter-meal intervals are primary determinants of food intake in the short-term, but macronutrient composition, particularly protein content and composition, may play a greater role for glycemic control [18]. It has been shown that compared to water, beverages containing energy, such as FJ, satisfy thirst, and are associated with higher fullness and reduced hunger rating and desire to eat [19]. Liquid carbohydrates have been reported to produce less satiety compared to solid carbohydrates, leading to an increase in total long-term energy intake [20,21]. One study reported that apple juice speeds gastric emptying and decreases postprandial intestinal volumes and satiety in healthy adults [22]. Another study examining preloads of apple in different forms prior to a meal, reported that apple juice compared to raw apple decreased the fullness sensation and satiety, and increased energy intake [23].

Therefore, the aims of this study were to investigate the short-term effects of a commercial regular mixed FJ (apple, orange, grape, and pomegranate), and the same FJ fortified with biofunctional ingredients (vitamin D3, n-3 polyunsaturated fatty acids (PUFA), and probiotics (10^8^ cfu/mL *Lacticaseibacillus casei* Shirota and *Lacticaseibacillus rhamnosus* GG)), either one by one or in combination, compared to a glucose drink on GI, postprandial glycemic responses and subjective appetite scores.

## 2. Materials and Methods

### 2.1. Participants

Healthy (clinically and metabolically) individuals (men and women) participated in this randomized controlled, double-blind, crossover, clinical trial. Volunteers became aware about this study from online advertisements, notices, and flyers posted around the university campus. Inclusion criteria for participation included a body mass index (BMI) value between 18 and 25 kg/m^2^, age between 18 and 55 years old, normal fasting blood glucose values (<100 mg/dL), and normal BP values [systolic blood pressure (SBP) < 120 mmHg and diastolic blood pressure (SBP) < 80 mmHg]. Exclusion criteria for participation included chronic diseases (e.g., CVD, diabetes mellitus, liver, or renal diseases), gastrointestinal disorders, pregnancy, lactation, polycystic ovary syndrome, attending competitive sports, and excessive alcohol consumption.

Volunteers underwent an initial screening before entering the study that included anthropometric measurements (height, body weight, waist and hip circumferences), body composition analysis via the bioimpedance method (InBody 230, Biospace, West Des Moines, IA, USA), fasting blood glucose measurements via the finger-prick method (calibrated MediSmart Ruby glucose meter with lancing device, Lilly Pharmaserv SA, Athens, Greece), BP measurements (Omron, Intellisense, HEM-907, Omron Hellas, Athens, Greece), and completion of a general health questionnaire. Twenty participants completed the initial evaluation, but eleven met all the criteria and were included for analyses.

The sample size calculation used the *t* distribution assuming an average coefficient of within-individual variation (CV) of the iAUC for blood glucose values of 25%. Ten volunteers would be required to achieve 80% power to detect a 33% between individual difference in iAUC with two-tailed *p* < 0.050. Eleven participants are required to achieve 80% power to detect a 1.33 mmHg between-individual difference in BP with two-tailed *p* < 0.050.

The study was conducted at the Laboratory of Dietetics and Quality of Life, Agricultural University of Athens, Greece. All participants gave their written consent for inclusion before participating in the study. The study protocol was approved by the Bioethics Committee of the Agricultural University of Athens (HRBD 78 12/10/2022) and was carried out in accordance with the Declaration of Helsinki [24]. ClinicalTrials.gov identifier: NCT05702359.

### 2.2. Study Design

**Figure 1:**
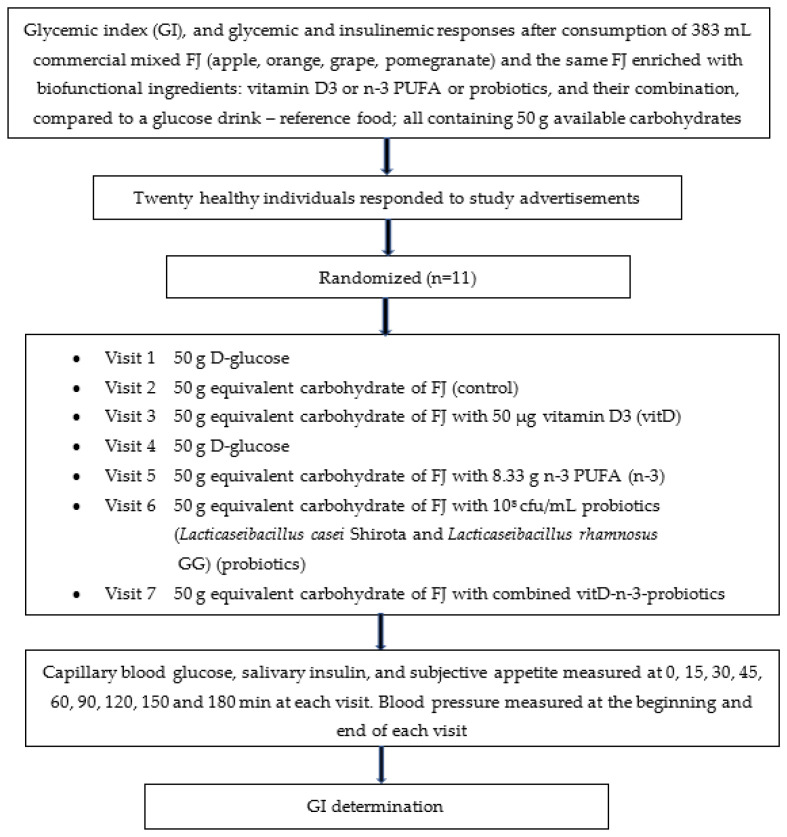
Flowchart of the study given as example of the randomized, crossover design. Participants were studied in separate days over a period of 3–9 weeks with an interval of no less than 40 h and more than 2 weeks between tests, and a wash-out period of at least two days in between visits. Abbreviations: FJ = fruit juice, GI = glycemic index.

The study’s design is described in Figure 1.

#### 2.2.1. Fruit Juice (FJ) Meals and Reference Drink

The FJ used in this study was a commercial pasteurized mixed FJ that was purchased from local supermarket (Olympos, S.A, Larissa, Greece) and stored under refrigeration. In the current study, four types of the same commercial FJ were prepared. Specifically, the four types of FJ were enriched by trained personnel from our team with one of the three biofunctional ingredients and with their combination: (i) vitamin D3 (vitD; dry vitamin D3 100 GFP SD, BASF SA, Ludwigshafen, Rhineland-Palatinate, Germany), or (ii) n-3 PUFA (n-3; dry n-3^®^ 12 food, dry powder consisting of spherical particles containing microencapsulated fish oil rich in eicosatetraenoic acid (302.5 g/mol) and docosahexaenoic acid (328.5 g/mol), BASF SA, Ludwigshafen, Germany), or (iii) probiotics (108 cfu/mL *Lacticaseibacillus casei* Shirota ACA-DC 6002 and *Lacticaseibacillus rhamnosus* GG ATCC 53103), or (iv) FJ enriched with the combination of the 3 aforementioned biofunctional ingredients (vitD-n-3-probiotics). All 4 FJs enriched with biofunctional ingredients were tested versus the control FJ and the reference glucose drink. All FJ types tested in the clinical trial contained 50 g available carbohydrates, a sufficient quantity of carbohydrates to produce acute clinically significant differences in postprandial glycemia within 180 min compared to the reference drink (D-glucose). The GIs of the 5 FJ test meals were evaluated. The GI was determined according to ISO 26642:2010 International Organization for Standardization methodology [6]. The trial consisted of 7 visits: glucose drink as reference food, tested two times, and the FJ test beverages tested once, in different weeks, with random sequence according to the recommended GI methodology (Figure 1). For the simple randomization of the sequence of the tested foods an online computer software (Social Psychology Network, Middletown, CT, USA) was used (http://www.randomizer.org; accessed on 2 March 2021) [25]. Two researchers not involved in the collection and analysis of the scientific data, were responsible for the randomization of the volunteers to the test drink study days and the double-blind condition (both the volunteers and the researchers collecting the data were not aware of the test drink provided and consumed).

Volunteers arrived at the Lab of Dietetics and Quality of Life at around 08:45–09:00 in the morning following an overnight fast of 10–14 h. Volunteers were asked to maintain stable dietary and exercise habits throughout their participation in this study. Participants were also asked to refrain from alcohol on the previous evening, from vigorous exercise on the morning of their visit to the lab and were only allowed to eat the provided foods throughout the test meal sessions. Compliance to the above-mentioned instructions were monitored in every visit collecting a 24 h dietary recall and the International Physical Activity Questionnaire (IPAQ) from every participant. In the case that a volunteer was feeling unwell or had not complied with the preceding experimental instructions, the session was rescheduled for another day. On each test occasion, participants were weighed to confirm body weight stability. Each visit consisted of a test meal that was consumed at a comfortable pace within 10 min, and 3 h post-meal blood glucose and salivary insulin measurements. All the tested FJ test meals and the reference glucose drink were given in portions containing 50 g available carbohydrates (383 mL FJ and 50 g D-glucose). Test meals were served with 250 mL water in all 7 sessions.

The available carbohydrates were determined using the Megazyme assay kit (Megazyme kit-K-ACHDF, Megazyme Ltd., Scotland, UK), which calculates only absorbed carbohydrates (sugars and digestible starch), neglecting dietary fiber and resistant starch. The nutritional characteristics of the studied FJs are described in Table 1 and Table 2.

#### 2.2.2. Microbial Cultures and FJ Inoculation

The probiotic microorganisms *Lacticaseibacillus casei* Shirota (ACA-DC 6002) and *Lacticaseibacillus rhamnosus* GG (ATCC 53103) were used in the current study. Stock cultures were preserved at −80 °C in De Man, Rogosa and Sharpe (MRS) broth (MRS broth, 4017292, Biolife, Milano, Italy) supplemented with glycerol (70:30). The monocultures were revived by adding 100 μL of stock culture of each strain in 10 mL MRS broth and incubated at 30 °C for 24 h. A subculture of each strain was prepared in fresh MRS broth and incubated at 30 °C for 24 h. For juice inoculation, the fresh cultures were harvested by centrifugation (5000× *g*, 10 min, 4 °C), washed twice with sterile phosphate-buffered saline (PBS) solution (Oxoid Phosphate Buffered Saline Tablets, BR0014G, Thermo Fischer Scientific, Waltham, MA, USA) and then the pellet was resuspended in PBS solution. The population of each strain in the PBS solution was enumerated and estimated at 9 log cfu/mL. Equal volume of each strain (*Lacticaseibacillus casei* Shirota and *Lacticaseibacillus rhamnosus* GG) was added in 1 L of fresh juice under aseptic conditions (laminar cabinet) to achieve a final mixed population of 8 log cfu/mL in the juice matrix. The verification of inoculum was estimated after pour-plating the dilutions on MRS ISO Agar (4017282, Biolife, Milano, Italy).

### 2.3. GI Determination and Blood Glucose Concentrations

On each visit and at all time points, blood samples for glucose measurements were taken using the finger-prick method. Two fasting blood samples were obtained at 5 min intervals (−5, 0) and the average value of these two time points was taken to be the baseline (fasting) concentration. Participants were then served the test drink. Additional finger-prick blood glucose samples were collected at 15, 30, 45, 60, 90, 120, 150, and 180 min after they started to drink. Each blood glucose time value was the mean of two blood samples from the same drop of blood of each volunteer. Before and during a test session, a blood glucose test record was filled out with the participant’s initials, identification number, date, body weight, provided test drink, water consumption, time of drinking initiation, time of test drink completion, and any out of the ordinary activities. During the 180 min test, participants remained seated quietly. After the last blood or salivary sample had been obtained, participants were offered a snack and were informed that the test session was completed.

Capillary blood glucose was measured using calibrated glucometers with dehydrogenase-FAD test strips (Ruby test strips, Lilly Pharmaserv S.A., Athens, Greece). The repeatability and within-laboratory coefficient variations were 2%. The average blood glucose response curve was plotted by calculating the mean blood glucose concentrations of all volunteers at each time point. Then, for each sample and each study participant, the incremental area under the curve (iAUC) was calculated geometrically, using the trapezoid rule, and ignoring the area beneath the baseline. The GI calculation for each test drink sample used the method referred to as the mean of the ratios. For each participant, the ratio between the individual iAUC after consuming the test drink and the iAUC for the same participant after consuming the reference glucose drink was calculated and expressed as a percentage value. Then, the GI of each test drink was calculated as the average value of the ratios across all participants consuming the test drinks. The peak blood glucose, defined as the highest recorded blood glucose value minus the baseline value, and the peak blood glucose time, defined as the time elapsing from the start of a meal to the highest recorded blood glucose value, were calculated.

### 2.4. Salivary Insulin Concentrations

To determine salivary insulin concentrations, salivary samples were collected using the Salivette method (Sarstedt AG and CO, Nümbrecht, Germany) at baseline and at 15, 30, 45, 60, 90, 120, and 180 min after each test drink ingestion. Before the collection of the samples, participants washed their mouths with clear water to avoid food contamination. Then, they were asked to remove the cotton from the tube and press it with their tongue for approximately 1 min to collect saliva from all glands. The tubes were centrifuged (3000× *g* for 5 min) and stored at −80 °C. Salivary insulin levels were determined using a Human Insulin ELISA kit (ALPCO, 80-INSHU-E10.1, Salem, NH) based on a sandwich-type enzyme linked immunosorbent method. Although the ultrasensitive insulin ELISA kit is not indented to measure insulin in saliva, we tested it before its use and compared it to plasma samples and the results were satisfactory.

### 2.5. Blood Pressure (BP)

SBP and DBP were measured at the screening stage and at the beginning and end of each test drink session using an upper arm digital BP monitor (Omron HEM-907, Omron Hellas, Athens, Greece) in a quiet, warm setting. Participants remained seated quietly and rested for at least 5 min in the supine position with their arm supported at the level of the heart and three BP measurements were taken by an already introduced member of our trained research team to avoid the “white coat effect”, at 1 min intervals, with the three readings averaged.

### 2.6. Subjective Appetite Ratings

Participants rated their hunger, desire to eat, perceived fullness, thirst, preoccupation with food, and pleasure of eating the consumed test food, after eating on a horizontal line visual analogue scales (VAS), with a line length of 10 cm, a line width of 3 desktop publishing points, was black, had flat line endpoints, had an ascending numerical order of scale endpoints [i.e., “0” and “10”, for example neither hungry (0 mm), full (100 mm) or have desire for food in the middle (50 mm)], and used “0” and “10” as numerical anchors below the left and right endpoints, respectively [26]. VAS were given in the form a booklet, one scale per page [27]. VAS ratings were obtained at 0, 15, 30, 45, 60, 90, 120, 150, and 180 min after consumption of each test drink.

### 2.7. Dietary Intake

Dietary intake was assessed via 24 h recalls at every visit by a trained member of our research team, and analyzed using the Diet Analysis Plus program, as well as using the Hellenic and European Food Composition Databases (http://www.eurofir.org/foodinformation/food-composition-databases-2/; accessed on 1 March 2021). The databases were modified to include new foods and recipes. The purpose of collecting dietary intake was to confirm that participants refrained from changing their eating habits until the study was completed.

### 2.8. Statistical Analysis

Data were entered into a spreadsheet by two different individuals with the values compared at all time points to assure accurate transcription. iAUC was calculated for capillary blood glucose, salivary insulin, and subjective appetite scores, ignoring the area below baseline/fasting. For the purposes of AUC calculation, fasting capillary blood glucose was taken to be the mean of the first measurement of the blood glucose concentrations at times −5 min and 0 min. The GI was calculated by expressing each participant’s iAUC for the test meal as the percentage of the same participant’s mean iAUC for the two reference D-glucose drinks. Values were to be excluded if found to have more than 2 standard deviations (SD) above the mean. The mean within-individual coefficient of variation of glycemic responses elicited by repeated tests of oral glucose (termed reference CV) for each volunteer was calculated; namely the mean SD and CV (100 × SD/mean) of the blood glucose iAUC values elicited by the two repeated tests of 50 g D-glucose. Data are presented as means and standard error of the mean (SEM), unless stated otherwise. Statistical tests or data analysis were performed according to data distribution (tested by P-P and kernel density plots). Normality of data was tested using the Shapiro–Wilk test. Baseline differences for normally distributed continuous variables were evaluated using Student’s t-test. The Kruskal–Wallis test was used for skewed continuous data. Pearson’s chi square test was performed to determine group differences for categorical variables. Pearson’s *r* was used for correlations between normally distributed continuous variables. For non-normal distributions, correlation coefficients were calculated using Spearman’s rho and Kendall’s Tau. Glycemic load (GL) was calculated using the formula: GL = GI × g of available carbohydrate in a typical FJ serving (250 mL)/100. Between test drinks, ANOVA for a 2 × 2 crossover study was conducted for capillary blood glucose, salivary insulin, BP, and subjective appetite scores, assuming as per data results a fixed error. In a 2 × 2 design, we assume that there are no individual effects since a complete randomization process was followed for treatment allocation. The models included the factors “participant” (id), “sequence” for inter-subject variation, and “period” and “treatments” to account for intra-participant variability. Time × test food interaction was evaluated. Multiple comparisons between the interventions were tested post hoc using Tukey test with the Bonferroni correction. For all other parameters, one-way ANOVA was used to examine differences between test drinks followed by post hoc Tukey test and the Bonferroni correction. The evaluation of the test drinks on postprandial glycemia, insulin concentrations, and subjective satiety compared to control was further explored using linear mixed models for repeated measures analyses. Cofactors used in analyses included age, gender, body mass index, body fat (kg), and fat free mass (kg). Statistical significance was determined to be *p* < 0.050. All analyses were performed using SPSS software (version 23.0, SPSS Inc., Chicago, IL, USA).

## 3. Results

### 3.1. Participants’ Baseline Characteristics

The participants’ baseline characteristics are shown in Table 3. There were no intermittent missing values or dropouts.

### 3.2. GI οf Test Drinks

Individual GI values were tested for outliers (values higher than two SD points) [6]. No outliers were found. The GI and GL values for the FJs are presented in Table 4. All test FJs with and without the addition of biofunctional ingredients are categorized as low GI foods. Compared to the reference glucose drink, all five FJs had significantly lower GI values, without significant differences between them (Table 4). All five FJs are classified as medium GL foods (GL > 10 but <20, per 250 mL FJ serving) and provided significantly lower GL values compared to D-glucose, without significant differences between them (Table 4).

No significant differences were observed in fasting blood glucose values between the tested FJs and the reference drink (D-glucose) (*p* for all > 0.05). Compared to the reference glucose drink, lower blood glucose concentrations were observed as changes from baseline at 15 min after the consumption of FJ combination (*p* = 0.02), n-3 (*p* = 0.009), and probiotics (*p* = 0.05). Compared to the reference glucose drink, lower blood glucose concentrations were observed as changes from baseline after the consumption of all five FJs types at 30 min (FJ combination: *p* = 0.002; n-3: *p* = 0.003; vitD: *p* = 0.01; probiotics: *p* = 0.03; and FJ control: *p* = 0.02), 45 min (FJ combination: *p* < 0.001; n-3: *p* < 0.001; vitD: *p* < 0.001; probiotics: *p* < 0.001; and FJ control: *p* = 0.001), 60 min (*p* for all < 0.001); and 90 min (FJ combination: *p* = 0.03; n-3: *p* = 0.006; vitD: *p* = 0.02; probiotics: *p* = 0.01; and FJ control: *p* = 0.007). Compared to the reference glucose drink, lower peak for blood glucose values were observed as changes from baseline after the consumption of all five FJs with and without addition of biofunctional ingredients (FJ combination: *p* < 0.001; n-3: *p* < 0.001; vitD: *p* = 0.001; probiotics: *p* = 0.001; and FJ control: *p* = 0.006; Table 4). No significant differences were found between test beverages and the reference food for time to peak values for blood glucose (*p* for all > 0.05). The average intra-participant coefficient variation of iAUC after the two repeated D-glucose tests was 25%. The 0–120 min iAUC for blood glucose values calculated as changes from baseline for all five FJs were significantly lower than those of the reference glucose drink (FJ combination: *p* < 0.001; n-3: *p* < 0.001; vitD: *p* < 0.001; probiotics: *p* < 0.001; and FJ control: *p* = 0.001; Table 4).

#### 3.2.1. Blood Glucose Concentrations Comparing the Tested FJ with Control FJ

**Figure 2:**
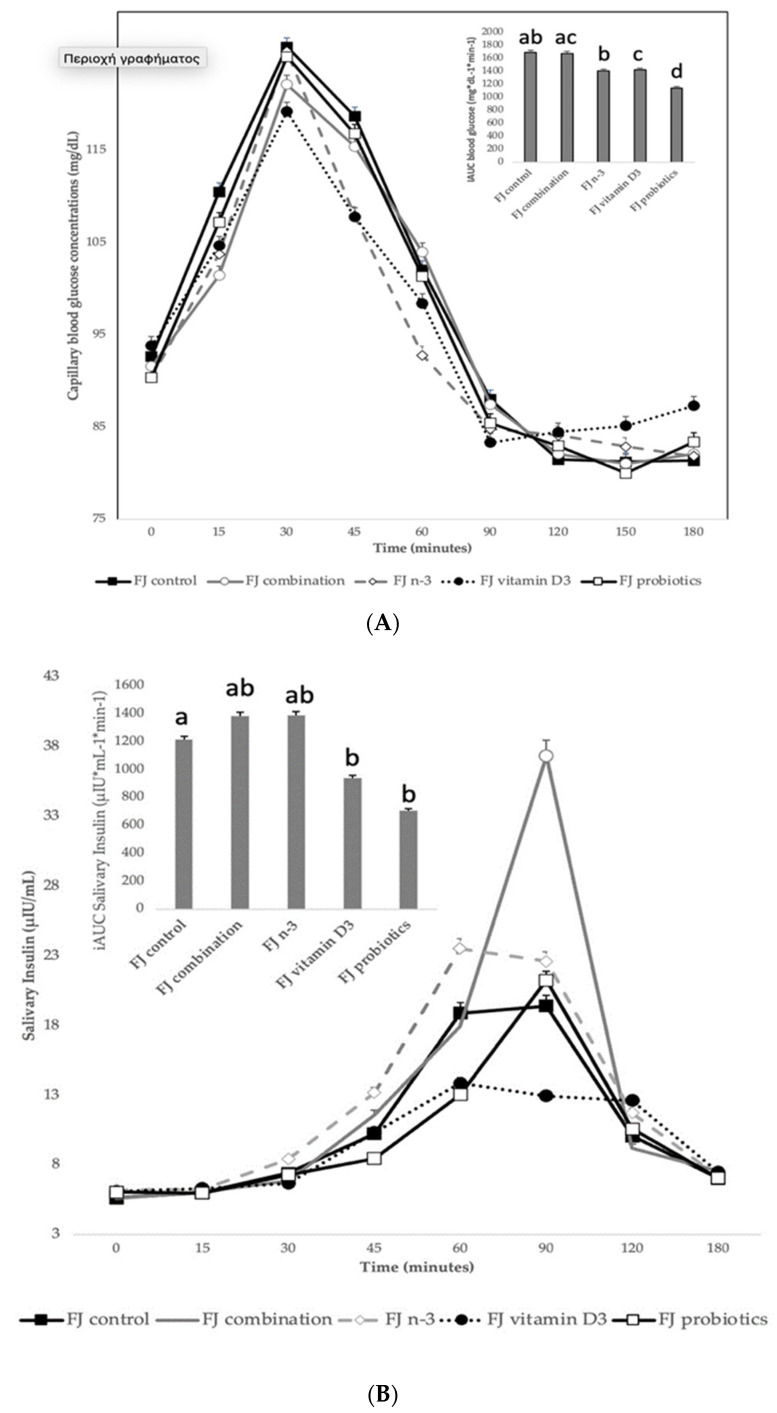

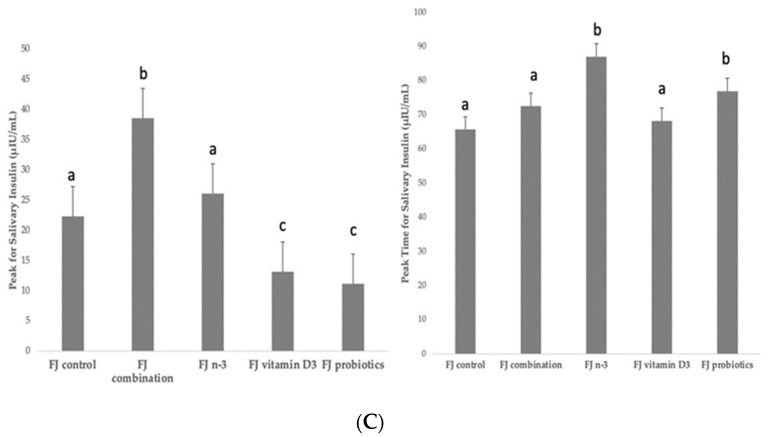
(**A**) Data are expressed as means ± SEM. Blood glucose concentrations (mg/dL) after the consumption of the tested fruit juices (FJ)s: FJ control, FJ with added vitamin D3, FJ with added n-3 PUFA, FJ with added probiotics, and FJ combination with added vitamin D3, n-3 PUFA, and probiotics, all containing 50 g available carbohydrates (n = 11). Values labeled with different superscript letter are significantly different (*p* < 0.05). (**B**) Data are expressed as means ± SEM. Salivary insulin concentrations (μU/mL) and iAUC for salivary insulin concentrations after the consumption of the tested fruit juices (FJ)s: FJ control, FJ with added vitamin D3, FJ with added n-3 PUFA, FJ with added probiotics, and FJ combination with added vitamin D3, n-3 PUFA and probiotics, all containing 50 g available carbohydrates (n = 11). Values labeled with different superscript letter are significantly different (*p* < 0.05). (**C**) Data are expressed as means ± SEM. Peak values and peak time values for salivary insulin concentrations (μU/mL) after the consumption of the tested fruit juices (FJ)s: FJ control, FJ with added vitamin D3, FJ with added n-3 PUFA, FJ with added probiotics, and FJ combination with added vitamin D3, n-3 PUFA, and probiotics, all containing 50 g available carbohydrates (n = 11). Values labeled with different superscript letter are significantly different (*p* < 0.05).

Figure 2A describes the blood glucose response curves (mg/dL) and the iAUC for capillary blood glucose concentrations after the consumption of the tested enriched FJs and the control FJ. No significant differences were observed in fasting blood glucose values between the tested FJs (*p* for all > 0.05). There was a significant blood glucose × time × test drink interaction (*p* = 0.009), a glucose × time × gender interaction (*p* = 0.001), a glucose × time × age interaction (*p* = 0.04), a glucose × time × fat free mass interaction (*p* = 0.009), a main effect of test drink on blood glucose concentrations (*p* <0.001) and a main effect of age (*p* = 0.003). Compared to control FJ, lower blood glucose concentrations were observed for FJ combination at 15 min post-test drink ingestion (*p* < 0.001). Compared to control FJ, significantly lower blood glucose concentrations were observed for FJ with n-3 at 15, 60, 90, and 120 min post-test drink ingestion (*p* = 0.01, *p* = 0.008, *p* = 0.02, and *p* = 0.001, respectively). Compared to FJ control, significantly lower blood glucose concentrations were observed for FJ with vitamin D at 15 and 90 min postprandially (*p* = 0.003 and *p* < 0.001, respectively), but higher blood glucose concentrations at 150 and 180 min postprandially (*p* = 0.03 and *p* < 0.001, respectively). At 60 min post-test drink ingestion, lower blood glucose concentrations were observed for FJ with n-3 compared to FJ combination (*p* < 0.001).

There was a main effect of test drink on iAUC for blood glucose (*p* = 0.02). Compared to the control FJ, FJ with probiotics had significantly lower iAUC for blood glucose (*p* < 0.001) and the FJ with vitamin D tended to have lower iAUC for blood glucose (*p* = 0.053). Compared to FJ combination, the FJ with n-3 and the FJ with probiotics had significantly lower iAUC for blood glucose (*p* = 0.05 and *p* < 0.001, respectively).

#### 3.2.2. Salivary Insulin Concentrations Comparing the Tested FJ with Control FJ

Figure 2B describes the salivary insulin response curves (μU/mL) after the consumption of the test FJs and the control FJ. There was a significant salivary insulin x time interaction (*p* = 0.04) and a significant iAUC for salivary insulin × time × meal interaction (*p* < 0.001). There was also a significant insulin × age interaction (*p* = 0.01), an insulin x BMI interaction (*p* = 0.049), and an insulin × fat mass interaction (*p* = 0.01), indicating a main effect of age in insulin concentrations (*p* < 0.001), of BMI (*p* = 0.004) and of fat mass (*p* < 0.001). Compared to the FJ control, lower salivary insulin concentrations were observed at 30 min post-test drink ingestion for FJ combination (*p* = 0.03) and FJ with vitamin D3 (*p* < 0.001), but higher for FJ with n-3 (*p* < 0.001). Compared to control FJ, significantly lower salivary insulin concentrations were observed at 90 min postprandially for FJ with vitamin D3 (*p* = 0.02), but significantly higher for FJ combination (*p* < 0.001). Compared to the FJ control, iAUC for salivary insulin concentrations was significantly lower for FJ with vitamin D and FJ with probiotics (*p* for both < 0.001). Compared to control FJ, significantly higher peak for salivary insulin was observed for FJ combination (*p* < 0.001) and it also tended to be higher for FJ with n-3 (*p* = 0.06) and significantly lower peak for salivary insulin was observed for FJ with vitamin D3 and FJ with probiotics (*p* for both < 0.001; Figure 2C). Compared to control FJ, significantly higher time to peak for salivary insulin was observed for FJ with n-3 (*p* = 0.001) and for FJ with probiotics (*p* = 0.02; Figure 2C).

### 3.3. Blood Pressure (BP)

There was a significant SBP x gender interaction (*p* = 0.01), a significant SBP x BMI interaction (*p* = 0.01), a SBP x fat mass interaction (*p* = 0.001), and a SBP × fat free mass interaction (*p* < 0.001). No differences were observed between the control FJ and the tested FJs (*p* for all > 0.05). There was only a trend for increased SBP at the end of the intervention compared to the beginning for FJ control, FJ with n-3, and FJ with vitamin D3 (*p* for all = 0.06). No differences were observed for DBP between the FJ control and the tested FJs or between beginning and end of test-drink ingestion.

### 3.4. Subjective Appetite Ratings

**Figure 3:**
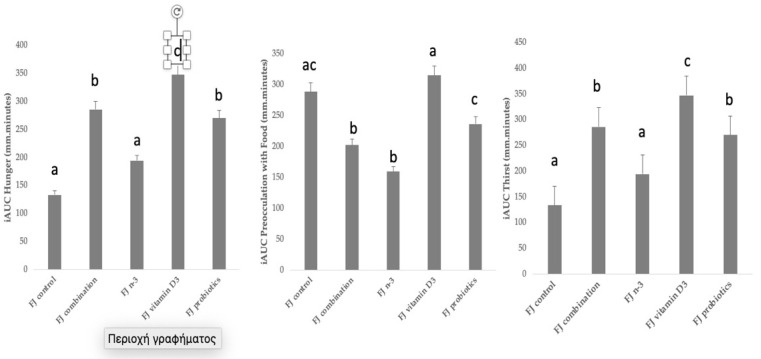
Data are expressed as means ± SEM. Incremental area under the curve for selected subjective appetite scores evaluated in visual analogue scales (VAS) after the consumption of the tested fruit juices (FJ)s: FJ control, FJ with added vitamin D3, FJ with added n-3 PUFA, FJ with added probiotics, and FJ combination with added vitamin D3, n-3 PUFA, and probiotics, all containing 50 g available carbohydrates (n = 11). Values labeled with different superscript letter are significantly different (*p* < 0.05).

Figure 3 describes the incremental area under the curve (iAUC) for selected subjective appetite ratings. Compared to control FJ, significantly higher iAUC for hunger was observed after the consumption of FJ combination, FJ with vitamin D3, and FJ with probiotics (*p* = 0.001, *p* < 0.001, and *p* = 0.001, respectively), and it only tended to be higher after FJ with n-3 (*p* = 0.054). Compared to control FJ, significantly lower iAUC for preoccupation with food was observed after the consumption of FJ combination (*p* = 0.03) and FJ with n-3 (*p* = 0.04). Compared to control FJ, significantly higher iAUC for thirst was observed after the consumption of FJ combination (*p* = 0.001), FJ with vitamin D3 (*p* < 0.001), and FJ with probiotics (*p* = 0.001), and it only tended to be higher after FJ with n-3 (*p* = 0.054). No differences between FJ control and the test FJs were observed for iAUC for feeling of fullness, iAUC for motivation to eat, and iAUC for pleasure (*p* for all > 0.05). Cofactors, such as age, gender, BMI, fat mass, and fat free mass, did not significantly affect the subjective appetite results.

## 4. Discussion

By applying the standard GI methodology this study produced novel data for the acute effects of FJ (apple, orange, grape, and pomegranate) consumption (FJ control) and the same FJ enriched with biofunctional ingredients: vitD, or n-3, or probiotics, or FJ combination (vitD, n-3, and probiotics) on postprandial glycemic and salivary insulin responses, BP, and subjective satiety scores. The results showed that the glycemic responses of the five tested FJ types were significantly lower than the responses of the reference glucose drink. Compared to the control FJ, FJs with n-3, vitamin D3, and probiotics led to reduced iAUC for blood glucose concentrations, which were partly explained by differences in salivary insulin levels and peak values for insulin concentrations.

### 4.1. GI and Glycemic Responses: Biofunctional Ingredients’ Implications

The current investigation showed that a mixed commercial FJ (apple, orange, grape, and pomegranate) without (control FJ) and enriched with vitamin D3 or n-3 PUFA or two alive probiotic strains or with the combination of vitamin D3 and n-3 PUFA and alive probiotics, produced similarly low GI foods, with significantly lower blood glucose peaks and blood glucose excursions when compared to the reference glucose drink.

These results indicate that the addition of biofunctional ingredients, i.e., vitamin D3, or n-3 PUFA, or probiotics, or their combination does not induce acute changes in the GI of tested FJs. Our results are in agreement with the international tables for GI and GL values, reporting GI values of 44 to 46 for apple juice, mixed apple, orange, and pineapple juice with a GI value of 47, grape juice from 52 to 63, and pomegranate juice with a GI value of 53 [15,28]. One study reported that pomegranate juice lowered the glycemic response in a high GI food, while the microbial metabolites from pomegranate polyphenols exhibited the potential to further modulate sugar metabolism much later in the postprandial period [29], which may partially explain the lower postprandial glucose concentrations observed also in our study. Our results are also partially in agreement with a study examining the effects of orange juice consumption, fresh or processed, compared to an isocaloric control beverage on postprandial blood glucose and insulin concentrations in lean and obese individuals, reporting that only in lean participants, orange juice compared to control beverage led to a reduction in the total concentration of blood glucose and insulin; whereas in obese participants, only a lower glucose peak was observed at 60 min after orange juice consumption compared to control beverage, without a significant reduction in total blood glucose and insulin levels [30].

Our results are in partial agreement with one study reporting that co-supplementation of probiotics with n-3 PUFA or placebo for 8 weeks led to a modest improvement in insulin resistance in people with T2DM compared to placebo [31]. Our results may be partially explained by the ability of n-3 PUFA to activate AMP-activated protein kinase, known to reduce lipogenesis, increase the oxidation of fatty acids, and stimulate non-insulin-regulated glucose transport into the cell, in white adipose tissues, muscles, and the liver, increase insulin sensitivity, and decrease adipocyte proliferation, thus resulting in improved glucose and insulin metabolism [32].

Our results may also be partially explained by the ability of probiotic ingestion to influence intestinal bacteria composition leading to modest improvement of carbohydrate and lipid metabolism due to increased glucagon like peptide-1 secretion, suppression of the toll like receptor-4 signaling pathway, and modulation of the peroxisome proliferator-activated receptor gamma (PPAR-gamma), known to improve fatty acid metabolism and insulin sensitivity [33]. Moreover, our results may be partially explained by the potent alpha-glucosidase inhibitory activity, an enzyme known to delay the digestion and absorption of carbohydrates, reducing postprandial hyperglycemia, exhibited mainly by *Lacticaseibacillus casei* and *Lacticaseibacillus rhamnosus* GG [34], the strains used in this study.

Vitamin D has been shown to activate the insulin receptor synthesis gene by binding to the nuclear receptor leading to increased presence of insulin-dependent glucose transporter type-4 in the cell membrane, enhanced beta-cell function via a vitamin D receptor-dependent manner, and increased expression of PPAR-gamma activated receptor gene [35,36]. It has been proposed that low baseline levels of 25-(OH)-vitamin D may be an independent predictor of insulin resistance [37]. In contrast, two studies have reported that vitamin D supplementation had no effect on insulin sensitivity or secretion, beta-cell function or glucose homeostasis, indicating that vitamin D3 supplementation is an ineffective strategy for lowering diabetes risk [38], even in vitamin D deficient populations [39]. However, results from a meta-analysis showed that fortification of foods, such as yogurt, with vitamin D may aid in weight loss and body fat loss and ameliorate blood glucose and blood lipids levels [40]. In conclusion, our results indicate that consumption of n-3 PUFA, or vitamin D3 or probiotics may lead to ameliorated postprandial glycemic responses.

### 4.2. Blood Pressure

Our results showed that SBP tended to increase slightly but not clinically significantly after the consumption of FJ with n-3, FJ with vitamin D3, and FJ control, which needs to be further evaluated. Overall, FJs without and enriched with bio-functional ingredients did not significantly affect SBP or DBP, at least acutely in normotensive, healthy participants.

### 4.3. Subjective Appetite Ratings

Results from the current investigation showed that biofunctional ingredients had a significant acute impact on subjective appetite ratings. Increased hunger ratings were observed after the consumption of FJ combination, FJ with vitamin D3 and FJ with probiotics. Lower preoccupation with food was observed after FJ with n-3 PUFA and FJ combination. Increased thirst was observed after the consumption of FJ combination, FJ with n-3 PUFA, and FJ with probiotics. Results from this study showed for the first time that addition of biofunctional ingredients to FJs acutely affected subjective satiety measures in different ways, independently of the FJ’s liquid carbohydrates. Our results are partially in agreement with a study examining the postprandial effects of a dose of orange juice, fresh or processed, in lean and obese individuals, reporting that both orange juices led to higher satiation in all participants [30].

It has been shown that vitamin D3 stimulates leptin and ghrelin secretion in a vitamin D receptor dependent manner, indicating that it may increase satiety and decrease energy consumption [41,42]. In addition, a high insulin level has been suggested to increase liver metabolism of nutrients, thus promoting satiety [43]. In this study, FJ with vitamin D3 and FJ with probiotics led to lower salivary insulin secretion, which may partially explain the higher feeling of hunger observed in our participants.

It has been shown that probiotics, particularly *Lactobacillus rhamnosus* GG, can decrease circulating leptin levels by altering the gut microbiota, increasing the ratio of villus height to crypt depth, and decreasing the proportion of Proteobacteria in fecal microbiota [44,45]. Our results are in partial disagreement with others reporting that *Lacticaseibacillus rhamnosus* increased significantly more insulin levels compared to control after 3 h [46]. Our results are also in partial disagreement with others reporting that prebiotics, probiotics, or their combination do not produce acute significant differences in hunger satiety scores or in the short-term in healthy men [47].

The role of n-3 PUFA in satiety and energy metabolism is not yet clear. Some suggest that n-3 PUFA increase energy intakes, but others support that n-3 PUFA suppress reward-seeking behaviors; all indicating that there may be a role for n-3 PUFA in overall energy intake regulation managing both over and under consumption [48]. In agreement with our results, one study showed that n-3 intake (2000 mg/day; 360 mg EPA, 240 mg DHA) for 4 weeks decreased appetite [49], and another study showed that consumption of a fish meal containing 3.65 g n-3 decreased total energy intake [50]. In contrast to our findings, one double-blind placebo control RCT assessing the effects of a 3-week n-3 PUFA supplementation (2000 mg/day; EPA: 360 mg, DHA: 240 mg) on food intake and appetite in young male athletes with normal body fat percentage, reported that carbohydrate intake, hunger, desire to eat, and desire to eat sweet foods increased with n-3 PUFA, and satiety decreased [51]. Also, contrary to our results, four studies reported that n-3 supplementation daily for 4 up to 12 weeks did not result in any significant change in subjective appetite scores and had no effects on total energy and macronutrient consumption [52,53,54,55]. Differences in population under study, health condition of participants, source of biofunctional ingredients (i.e., n-3 as supplement or food) and varying doses, duration of dietary intervention, and physical activity status may be some reasons for the conflicting study results.

### 4.4. Limitations and Advantages

The strength of our study includes the randomized, crossover, double-blind design where each participant served as his/her own control. The major limitation of the current study is the acute feeding protocol, which does not allow conclusions for long-term benefits. Another limitation may be the small sample size, although the results were consistent with low diversity and the lack of measurement of plasma vitamin D levels of participants. Moreover, our study was conducted in healthy, normoglycemic, and normotensive young adults with normal weight, and therefore our results need to be confirmed in other populations, i.e., middle-aged or elderly people with prediabetes or T2DM with and without obesity. In addition, our results need to be confirmed in RCTs of longer duration.

### 4.5. Practical Applications

To the best of our knowledge, this trial determined, for the first time, the short-term effects of FJ consumption without and with added vitamin D3, or n-3 PUFA, or two alive strains of probiotics, or with the combination of these biofunctional ingredients, on glycemic and insulinemic responses, BP, and subjective appetite scores. Our results showed that FJs with added biofunctional ingredients produced acute differences in glycemic and insulinemic measurements and subjective appetite scores and that consumption of FJ control and FJ enriched with biofunctional ingredients produced low GI values and lower blood glucose excursions, relative to the reference glucose drink, indicating that it may be a suitable dietary choice for all individuals, including people with prediabetes or diabetes mellitus, which needs to be confirmed in longer duration studies and in high cardiometabolic risk populations.

## 5. Conclusions

In conclusion, FJ with added biofunctional ingredients, i.e., vitamin D3 or probiotics or n-3 PUFA or the combination of all these three ingredients provided low GI food values. It was interesting to observe that compared to the control FJ and FJ combination, consumption of FJ with n-3, vitamin D3, and probiotics led to ameliorated postprandial glycemic responses, which were partly explained by differences in salivary insulin levels and peak values for insulin concentrations. In addition, compared to the FJ control, consumption of the FJ with vitamin D3, or probiotics, or with the combination of n-3 PUFA, vitamin D3, and probiotics increased hunger, decreased preoccupation with food, and increased thirst, which needs to be further investigated. Future long-term studies are needed to confirm these results and provide a long-term insight regarding the mechanism by which a FJ with biofunctional ingredients may or may not elicit a favorable impact on glycemic responses.

## Figures and Tables

**Table 1:** Nutrient composition of mixed fruit juice (apple, orange, grape, and pomegranate) per 100 mL based on the food label.

Per Serving (100 mL)	Fruit Juice
Energy (kcal/kJ)	51/211
Fat (g)	0.1
Saturated fat (g)	0.0
Total Carbohydrates (g)	12.1
Sugars (g)	12.1
Protein (g)	0.3
Sodium (mg)	0.0
* Available Carbohydrates (g)	13.1

* The available carbohydrates were determined using the Megazyme assay kit (Megazyme kit-K-ACHDF, Megazyme Ltd., Scotland, UK).

**Table 2:** Amounts of biofunctional ingredients added to fruit juice.

Biofunctional Ingredients Added to Fruit Juice * per 383 mL (Equivalent to 50 g Available Carbohydrates)	
Dry vitamin D3 (μg/IU)	50/2000
Dry n-3 PUFA (g)	8.33 (EPA: 533 mg, DHA: 267 mg)
Probiotics (*Lacticaseibacillus casei* Shirota, *Lacticaseibacillus rhamnosus* GG) (cfu/mL)	10^8^

* pH = 3.6; Abbreviations: PUFA = polyunsaturated fatty acids; EPA = eicosapentaenoic acid; DHA = docosahexaenoic acid.

**Table 3:** Participants’ baseline characteristics (n = 11).

Characteristics	Total
N	11 (6 men, 5 women)
Age (years)	25 ± 2
Body weight (kg)	68 ± 4
Height (cm)	172 ± 3
Body mass index (BMI; kg/m^2^)	23 ± 1
Body fat (kg)	16 ± 2
Muscle mass (kg)	30 ± 2
Basal metabolic rate (kcal)	1509 ± 69
Waist circumference (cm)	80 ± 3
Hip circumference (cm)	96 ± 2
Dietary intake (from 24 h recall)	
Protein (g)	96 ± 8
Carbohydrate (g)	264 ± 21
Fat (g)	106 ± 7
Saturated fat (g)	30 ± 2
Total cholesterol (mg)	312 ± 55
Dietary fiber (g)	20 ± 2
Sodium (mg)	2648 ± 198
Energy intake (kcal)	2380 ± 162

Values are expressed as means ± SEM.

**Table 4:** Incremental area under the curve (iAUC) for blood glucose, glycemic index (GI), glycemic load (GL), and peak for blood glucose values of FJ control and FJ enriched with biofunctional ingredients, relative to D-glucose (reference food).

Meal (Serving Portion Containing 50 g Available Carbohydrates)	iAUC (mg·120 min·dL^−1^)	GI (D-Glucose as Reference Food)	GL (D-Glucose as Reference Food)	Blood Glucose Peak Value (mg/dL)
D-Glucose	2992 ± 298 ^a^	100 ^a^	-	55 ± 4 ^a^
FJ control	1577 ± 244 ^b^	54 ± 9 ^b^	18 ± 3 ^b^	38 ± 4 ^b^
FJ enriched with vitamin D3	1457 ± 160 ^b^	52 ± 6 ^b^	17 ± 2 ^b^	35 ± 3 ^b^
FJ enriched with n-3 PUFA	1324 ± 135 ^b^	51 ± 9 ^b^	17 ± 3 ^b^	34 ± 3 ^b^
FJ enriched with probiotics	1399 ± 206 ^b^	50 ± 8 ^b^	16 ± 2 ^b^	36 ± 4 ^b^
FJ combination (VitD-n-3-probiotics)	1504 ± 151 ^b^	52 ± 5 ^b^	17 ± 2 ^b^	34 ± 2 ^b^

Data are expressed as means ± SEM. Each value represents the mean of eleven participants. Values labeled with different superscript letter are significantly different (*p* < 0.05). Means were compared column-wise using one-way ANOVA for factor “treatment”, “period”, and “sequence of treatment”, and post-hoc Tukey test with the Bonferroni correction to account for multiple comparisons between test meals; *p*-values < 0.050 were accounted as significant. Abbreviation: FJ = fruit juice.

## Data Availability

Not applicable.
